# Mesenchymal stromal cells pretreated with proinflammatory cytokines enhance skin wound healing via IL-6-dependent M2 polarization

**DOI:** 10.1186/s13287-022-02934-9

**Published:** 2022-08-13

**Authors:** Chenyang Liu, Yan Xu, Yichi Lu, Pan Du, Xiaoxiao Li, Chengchun Wang, Peng Guo, Ling Diao, Guozhong Lu

**Affiliations:** 1 Nanjng University of Traditional Chinese Medcine, Nanjng, Jiangsu China; 2 Wuxi School of Medicine, Jiangnan University, Wuxi, Jiangsu China; 3 Nantong University, Nantong, Jiangsu China; 4 Engineering Research Center of the Ministry of Education for Wound Repair Technology, Jiangnan University, The Affiliated Hospital of Jiangnan University, Jiangsu, China

**Keywords:** Mesenchymal stromal cells, IFN-γ and TNF-α, M2 macrophages, IL-6-dependent signaling pathways, Wound healing

## Abstract

**Background:**

Numerous studies have shown that mesenchymal stromal cells (MSCs) promote cutaneous wound healing via paracrine signaling. Our previous study found that the secretome of MSCs was significantly amplified by treatment with IFN-γ and TNF-α (IT). It has been known that macrophages are involved in the initiation and termination of inflammation, secretion of growth factors, phagocytosis, cell proliferation, and collagen deposition in wound, which is the key factor during wound healing. In this study, we aim to test whether the supernatant of MSCs pretreated with IT (S-IT MSCs) possesses a more pronounced effect on improving wound healing and describe the interplay between S-IT MSCs and macrophages as well as the potential mechanism in skin wound healing.

**Methods:**

In the present study, we used a unique supernatant of MSCs from human umbilical cord-derived MSCs (UC-MSCs) pretreated with IT, designated S-IT MSCs, subcutaneously injected into a mice total skin excision. We evaluated the effect of S-IT MSCs on the speed and quality of wound repair via IT MSCs-derived IL-6-dependent M2 polarization in vivo by hematoxylin–eosin staining (H&E), immunohistochemistry (IHC), immunofluorescence (IF), Masson’s trichrome staining, Sirius red staining, quantitative real-time PCR (qPCR). In addition, the effect of S-IT MSCs on the polarization of macrophages toward M2 phenotype and the potential mechanism of it were also investigated in vitro by flow cytometry (FCM), enzyme-linked immunosorbent assay (ELISA), tube formation assay, and western blot analysis.

**Results:**

Compared with control supernatant (S-MSCs), our H&E and IF results showed that S-IT MSCs were more effectively in promoting macrophages convert to the M2 phenotype and enhancing phagocytosis of M2 macrophages. Meanwhile, the results of tube formation assay, IHC, Masson’s trichrome staining, Sirius red staining showed that the abilities of M2 phenotype to promote vascularization and collagen deposition were significantly enhanced by S-IT MSCs-treated, thereby accelerating higher quality wound healing. Further, our ELISA, FCM, qPCR and western blot results showed that IL-6 was highly enriched in S-IT MSCs and acted as a key regulator to induce macrophages convert to the M2 phenotype through IL-6-dependent signaling pathways, ultimately achieving the above function of promoting wound repair.

**Conclusions:**

These findings provide the first evidence that the S-IT MSCs is more capable of eliciting M2 polarization of macrophages via IL-6-dependent signaling pathways and accelerating wound healing, which may represent a new strategy for optimizing the therapeutic effect of MSCs on wound healing.

**Supplementary Information:**

The online version contains supplementary material available at 10.1186/s13287-022-02934-9.

## Introduction

Wound healing is a complex process and overlapping continuous process that depends on the presence of various types of cells, growth factors, cytokines and extracellular matrix elements. MSCs, as a source of tissue cells, have the potential for self-renewal and differentiation, and they can realize functional tissue repair through self-differentiation and paracrine signaling. Studies have shown that MSCs significantly accelerate the healing of acute wounds, diabetic ulcers, pressure ulcers and other chronic wounds in animals or humans [1, 2]. In addition, the supernatant of MSCs has been showed to accelerate wound healing [3]. However, MSCs or MSC supernatant does not show excellent effects in all cases [4–6], indicating that the beneficial effect of MSCs to promote wound repair needs further improvement. Our study found that the paracrine effect of MSCs is plasticity; that is, under the stimulation of certain inflammatory factors, MSCs themselves mass-produce cytokines, including cytokines that are capable of regulating the immune microenvironment and stimulating the repair effect of endothelial cells, fibroblasts and tissue precursor cells in wounds, such as interleukin-6 (IL-6), CCL2, vascular endothelial growth factor (VEGF), and fibroblast growth factor (FGF) [7–9]. This suggests that stimulation of MSC by acute inflammatory factors confers on MSC an enhanced ability to promote wound repair.

The effects of MSCs on immune cells are diverse. MSCs have been shown to inhibit the differentiation of monocytes to dendritic cells, suppress the maturation of dendritic cells, inhibit the production of IFN-ɣ by NK cells, and have strong immunosuppressive effects on T and B cells [10, 11]. While the mechanisms of MSC-mediated immunosuppressive effects are not fully understood, they may be mediated by some soluble factors produced by MSCs. Currently, the most widely recognized MSC-derived soluble mediators are prostaglandin E2 (PGE2), indoleamine 2,3-dioxygenase (IDO), nitric oxide (NO), IL-6, and IL-10 [10, 12, 13]. For example, MSCs inhibit T cell proliferation by producing chemokines and NO [14].

In general, macrophages (Møs) are the key cells that regulate the immune microenvironment in the process of wound repair. M1 and M2 macrophages are two differentiation patterns. Classically activated M1 macrophages are labeled by specific proteins, such as CD86, iNOS, and TNF-α, and they mainly show proinflammatory properties [15]. Alternatively activated anti-inflammatory M2 macrophages are labeled with specific proteins, such as CD206, CD163, and Arg-1, and they exert their anti-inflammatory function, which promotes vascularization and collagen deposition by secreting IL-10, IL-4, IL-13, VEGF, and other cytokines, ultimately accelerating wound healing [16, 17]. Therefore, promoting M2 polarization is important to regulate the microenvironment of the wound and accelerate wound healing. Studies have been reported that MSC-derived PEG2, IDO, IL-6, and miR-223 accelerate wound healing by promoting macrophage polarization toward the M2 phenotype [5, 18–22]. Moreover, researchers found that MSCs secreted high levels of NO, IL-6, and PGE2 after preconditioned with IL-1ß and IFN-ɣ, and their further research demonstrated that MSC-mediated macrophage polarization strongly depends on IL-6, whereas a minor role for NO and PGE2 [20]. In addition, among the relevant signaling pathways that promote macrophage polarization toward M2, activated STAT3 and STAT6 are key transcription factors for macrophage polarization toward the M2 phenotype [23, 24].

In the present study, we found that IL-6 secretion of MSCs pre-stimulated by 20 ng/mL each of IFN-γ and TNF-α (10,571.33 ± 149.36 pg/mL) was tens of thousands of times higher than that secretion of MSCs (67.89 ± 28.91 pg/mL). We investigated whether the effect of IT MSCs in promoting M2 polarization through IL-6-dependent signaling pathways is more significant than that of MSCs. Therefore, we compared the effect of the S-IT MSCs to that of the S-MSCs on promoting macrophage polarization to the M2 phenotype via IL-6-dependent signaling pathways. We found that the S-IT MSCs was superior as it accelerated vascularization and collagen deposition in the process of wound healing in a manner dependent on the preconditioning of MSCs with proinflammatory cytokines. Therefore, we hypothesized that pretreatment with inflammatory cytokines is a better strategy for the future application of MSCs in wound healing in the clinic.

## Materials and methods

### Human UC-MSCs

The human UC-MSCs used in this experiments were taken from human umbilical cords, and the method is previously described [7, 25]. And the Medical Ethics Committee of Affiliated Hospital of Jiangnan University approved all experimental procedures. Ethics Approval No:LS2021046. Briefly, after parental consent, healthy full-term delivery umbilical cords were obtained and transferred to a biosafety cabinet with sterile PBS within 4 h. Under aseptic conditions, Waldron's Jelly was isolated from the umbilical cord, then cut into small pieces, and transferred to 10-cm dishes, and Dulbecco’s modified Eagle’s medium (DMEM, Gibco) containing 15% FBS (Gibco), 10 ng/ml bFGF (PeproTech), and 100 mg/mL penicillin/streptomycin (Gibco) was used to cover the tissue pieces and then cultured in a humid 37 °C, 5% CO_2_ incubator. Medium replenishment was performed every 3 days, and nonadherent cells and tissues were removed after 14 days.

### Macrophages

Macrophages were extracted from C57BL/6 J mice (age 4–6 weeks) bone marrow, method as previously described [26]. Briefly, the isolated femur and tibia bones were transferred to a biosafety cabinet after 5 min in 75% alcohol and then washed twice in sterile PBS. Bone marrow was isolated from bones using a 1-mL syringe with DMEM/F12 (Gibco) containing 10% FBS (Gibco), penicillin/streptomycin (100 µg/ml) (Gibco), and 20 ng/mL M-CSF (PeproTech) and then cultured in a humid 37 °C, 5% CO_2_ Incubator for 7 days. Expression of the macrophage marker, F4/80 (PE/Cyanine7-labeled) antibodies (BioLegend), was verified by flow cytometry. Only macrophages with a purity greater than 95% were used for subsequent experiments.

### Endothelial cells

Primary HUVECs were from PromoCell. Cells were cultured in DMEM (Gibco) containing 10% FBS (Gibco) and penicillin/streptomycin (100 µg/ml) (Gibco) at 37 °C under 5% CO_2_, and cells were used to perform tube formation assays.

### Preparation of stromal cell supernatant

MSCs were cultured in 10-cm-diameter dishes, and they were stimulated with IT (20 ng/mL, PeproTech) for 24 h when they reached 90% confluence and then were washed three times with PBS. Ultimately, the supernatant was collected after removing cell debris by centrifugation at 350 g for 5 min.

MSCs that were transferred using specialized IL-6 small interfering RNA (IL6-3 sense, 5’-3’ GUG AAG CUG AGU UAA UUU AdTdT; IL6-3 antisense, 5’-3’ UAA AUU AAC UCA GCU UCA CdTdT; IL6-4 sense, 5’-3’ CAA AGA AUC UAG AUG CAA UdTdT; and IL6-4 antisense, 5’-3’ AUU GCA UCU AGA UUC UUU GdTdT) were carried out according to the manufacturer's protocol (TranSheepBio). Nonsilencing siRNA was used to eliminate interference from the siRNA itself or transfection reagent. After MSCs were coincubated with SiRNA IL-6 or SiNC for 24 h, MSCs were stimulated with IT for another 24 h, and then MSCs were cultured in new DMEM for 12 h after washed with PBS for three times. Ultimately, the supernatant was collected after removing cell debris by centrifugation at 350 g for 5 min. In addition, transfection efficiency was confirmed by ELISA.

The supernatant of macrophages was collected, after they were stimulated by the supernatant of stromal cells in each group for 24 h, and then they were washed three times with PBS and cultured in new DMEM for 12 h. The supernatant was collected after removing cell debris by centrifugation at 350 g for 5 min.

All the above supernatants were condensed by a 3-kDa ultrafiltration membrane at 3234 × *g* for 45 min, resulting in the supernatant being condensed to a tenth of their original volume, and the condensed supernatant was then stored at − 80 °C. In some experiments, recombinant human IL-6 protein (PeproTech) was added at a concentration of 100 ng/mL.

### Analysis of cytokine production by enzyme-linked immunosorbent assay (ELISA)

The concentrations of IL-6 (EK106/2-96, MultiSciences), IL-13(EK113-96, MultiSciences), and IL-4(EK104-96, MultiSciences) in the S-MSCs and S-IT MSCs as well as the concentrations of TNF-α (EK282/4-96, MultiSciences), IL-10 (EK210/4-96, MultiSciences), VEGF (EK283/2-96, MultiSciences), IL-13 (EK213/2-96, MultiSciences), and IL-4(EK204HS-96, MultiSciences) in macrophages after each group was pretreated were measured by ELISA according to the manufacturer's directions. The absorbance (450 nm) for each supernatant was analyzed by a microplate reader (Cytation5, Bio Tek) and was interpolated with a standard curve.

### Flow cytometry

After culturing with the supernatant of each group of stromal sells for 24 h, the macrophages were collected, fixed with Fixation Buffer (BioLegend), and permeabilized with Intracellular Staining Perm Wash Buffer (Bio Legend). Then adjusting the density of cells to 1 × 10^6^/ml, and the following monoclonal fluorescent antibodies were then added: F4/80-PE/Cyanine7, CD206-APC, CD86-PE, Arg-1-PE, and iNOS-FITC (BioLegend). Cells were then incubated in the dark at 4 °C for 30 min, washed twice with PBS, and resuspended. The expression of related antigens was detected by flow cytometry.

For the in vitro phenotypic switch of M1 to M2 macrophages, macrophages were prestimulated with 200 ng/ml LPS (Sigma) for 2 h to induce an M1 phenotype, and the supernatant of each group of stromal cells was then added. The rearrangement of M1 to M2 was detected by flow cytometry. Gating strategies for the flow cytometry experiments are as follows: Mononuclear cells were first circled in FSC-A and SSC-A, then adherens were removed in FSC-H and FSC-A, and individual cells were circled for subsequent experimental analysis.

### Phagocytosis of macrophages

Macrophages were inoculated in 96-well plates at a density of 1 × 10^5^ cells/well, and ells were then treated according to the above experimental grouping after adhered. After 24 h of stimulation, the supernatant of each group was removed, and 100 μl/well of dextran MW 4000 solution (1 mg/ml) labeled with FITC was added. Cells continue to be incubated in the cell incubator for 30 min. Then, cells were washed three times with 200 μl/well PBS. Finally, cells were fixed with 4% paraformaldehyde and were observed using an inverted fluorescence microscope.

### Tube formation assay in vitro

For in vitro tube formation assay, 50 µl/well of Matrigel (BD Biosciences) is added to a 96-well plate (Corning) and incubated for 30 min at 37 °C under sterile conditions to solidify it. Subsequently, HUVECs were inoculated at a density of 2 × 10^4^ cells per 100 µL. Additionally, the supernatant of each group of macrophages was added. After 12 h, the formation of the tube was captured under an optical microscope (Motic Corporation, China). The number of branches was analyzed by ImageJ software.

### Western blot analysis

Western blot analysis was implemented as previously mentioned [27]. The primary antibodies used in this experiment are as follows: total and phosphorylated STAT3 (S727) (p-STAT3) (Abcam); total and phosphorylated STAT6 (Y641) (p-STAT6) (Abcam); PPAR-γ (Abcam); IL4Rα (Signalway Antibody); and β-actin (Signalway Antibody). The secondary antibody was goat anti-rabbit IgG (HRP) (Signalway Antibody). The blots were analyzed using ImageJ software.

### Wound-healing model and treatment

Thirty 6–8-week-old C57BL/6 J female mice were purchased from Shanghai SLAC Laboratory (Shanghai, China) and housed in the Medical Laboratory Animal Center of Jiangnan University. The Animal Ethics Committee of Jiangnan University authorized all experimental procedures in this study. Animal Ethics Approval No: JN.No20210430c0641130[113].

A mouse skin excision wound healing model was established according to the previously described method [28]. Briefly, 7 days after adaptive feeding, 28 C57BL/6 J female mice weighing 20–25 g were anesthetized, and two circular holes of 6 mm in diameter were formed in the back of the mice. The mice were randomly divided into the following three groups, DMEM and S-SiNC-MSCs (n = 12), S-SiNC-IT MSCs (n = 6), S-SiIL-6-IT MSCs, and S-SiIL-6-IT MSCs-recIL-6 (n = 12). The mice were treated on alternate days (20 μL for each treatment) by injecting evenly into four points of the wound bed. Then cover the wound with 3 M film (1624 W, 3 M) followed by a layer of medical tape to protect the wound from dryness and infection. Take photographs of the wounds every other day after the injury. ImageJ software was used to count the areas of the wound.

On days 3, 7, and 10, the mice were euthanized via cervical dislocation, and samples were harvested. Half of the wound tissue was immediately frozen at − 80℃ for tissue qPCR, and the remaining half was fixed with 4% paraformaldehyde for tissue hematoxylin–eosin (H&E) staining, Masson’s trichrome staining, immunofluorescence (IF), immunohistochemistry (IHC), and Sirius red staining analysis.

### Quantitative real-time PCR

Tissue samples harvested on day 3 were immediately frozen at − 80℃ for later use. Briefly, 100 mg of tissue was added to a mortar with an appropriate amount of liquid nitrogen, quickly ground, and then 1 ml of TRIzol reagent was added to continue grinding until no significant mass remained, and RNA was extracted with an RNA kit according to the manufacturer’s instructions. The expression of IL-4Rα, PPAR-γ, and VEGF was measured using the 2^−△△^CT method after reverse transcription and amplification steps. The primer sequences are provided in Table 1.

**Table 1:** Sequences of primers used for qPCR analysis

	Forward	Reverse
GAPDH	TGACATCAAGAAGGTGGTGAAGCAG	GTGTCGCTGTTGAAGTCGAGGAG
VEGF	GGGCTCTTCTCGCTCCGTAGTAG	CCCTCTCCTCTTCCTTCTCTTCCTC
IL-4Rα	CTGAGGCTGCTGCTACGATGAC	GAGGTTGGCTTCTGGTGGTATTCC
PPAR-γ	AGCCCTTTACCACAGTTGATTTCTCC	GCAGGTTCTACTTTGATCGCACTTTG

### Hematoxylin–eosin staining (H&E) and immunohistochemistry (IHC)

On days 3, 7, and 10 after the first supernatant treatment, the mice were euthanized via cervical dislocation, and samples were harvested for tissue H&E staining and IHC analysis. Briefly, the tissues were fixed with 4% paraformaldehyde for 48 h and the tissues were then covered with paraffin before sectioning and histological analysis. Blocks were cut into 4-μm-thick sections and stained with H&E (YESEN).

To explore vascularization of the wound, the rate of CD31 (Abcam, China)-positive signals was detected by IHC. The tissue sections were first deparaffinized and rehydrated prior to boiling in a 100 °C Tris/EDTA buffer (pH 9.0) water bath for 25 min (Beyotime, China). An IHC kit (Absin, China) was then used according to the manufacturer’s instructions. The rate of CD31-positive signals was calculated by ImageJ software.

### Immunofluorescence (IF) analysis

Samples from the wound bed on day 3 were first dewaxed and rehydrated and then repaired antigen via boiling in a 100 ℃ citrate buffer water bath for 25 min. The tissue sections were blocked with immunohistochemical blocking solution (Beyotime, China) for 90 min. The primary antibodies used in this experiment were incubated at 4 ℃ overnight, as follows: CD86 (Signalway Antibody, USA) and CD163 (GeneTex, USA) and then incubated with the following secondary antibodies for 90 min at room temperature: Alexa 488-conjugated goat anti-rabbit IgG (Abcam, UK) and Alexa 594-conjugated goat anti-mouse IgG (Abcam, UK). The nuclei were stained with DAPI (YESEN, China). Images were acquired using a laser-scanning confocal microscope (Carl Zeiss LSM880, Germany).

### Masson’s trichrome staining

Masson’s trichrome staining (Sbjbio) was used to observe collagen deposition in the wound on days 3 and 10 according to the manufacturer's instructions. Images were acquired using an optical microscope (Motic Corporation, China).

### Sirius red staining

The ratio of type I/III collagen on day 10 was observed by Sirius red staining to evaluate the quality of wound repair according to the manufacturer's instructions. Images were acquired using a polarized light microscope (BX51, OLYMPUS, Japan).

### Statistical analysis

All results are expressed as the mean ± SD. The experiments were independently repeated three times. Comparisons were performed by one-way analysis of variance followed by Tukey's multiple comparison post hoc test. Statistical analysis was performed using SPSS 22.0 software. P values less than 0.05 were considered statistically significant.

## Results

### The S-IT MSC efficiently converts macrophages toward the M2 phenotype and suppresses polarization toward the M1 phenotype

It has been shown that MSCs promote polarization toward the M2 phenotype and suppress polarization toward the M1 phenotype via paracrine factors [5, 18–22]. To explore whether the beneficial effect of MSCs on the above function is strengthened by pre-stimulation with IT, we examined the effect of IT-pretreated or unpretreated human umbilical cord-derived MSCs (UC-MSCs) supernatants on macrophagess. The MSCs used in this study were assayed by flow cytometry using a series of surface markers for characterize MSCs populations (Additional file 1: Fig. S1A). Bone marrow differentiation-derived macrophages were characterized using F4/80 (Additional file 1: Fig. S1B).

**Fig. 1:**
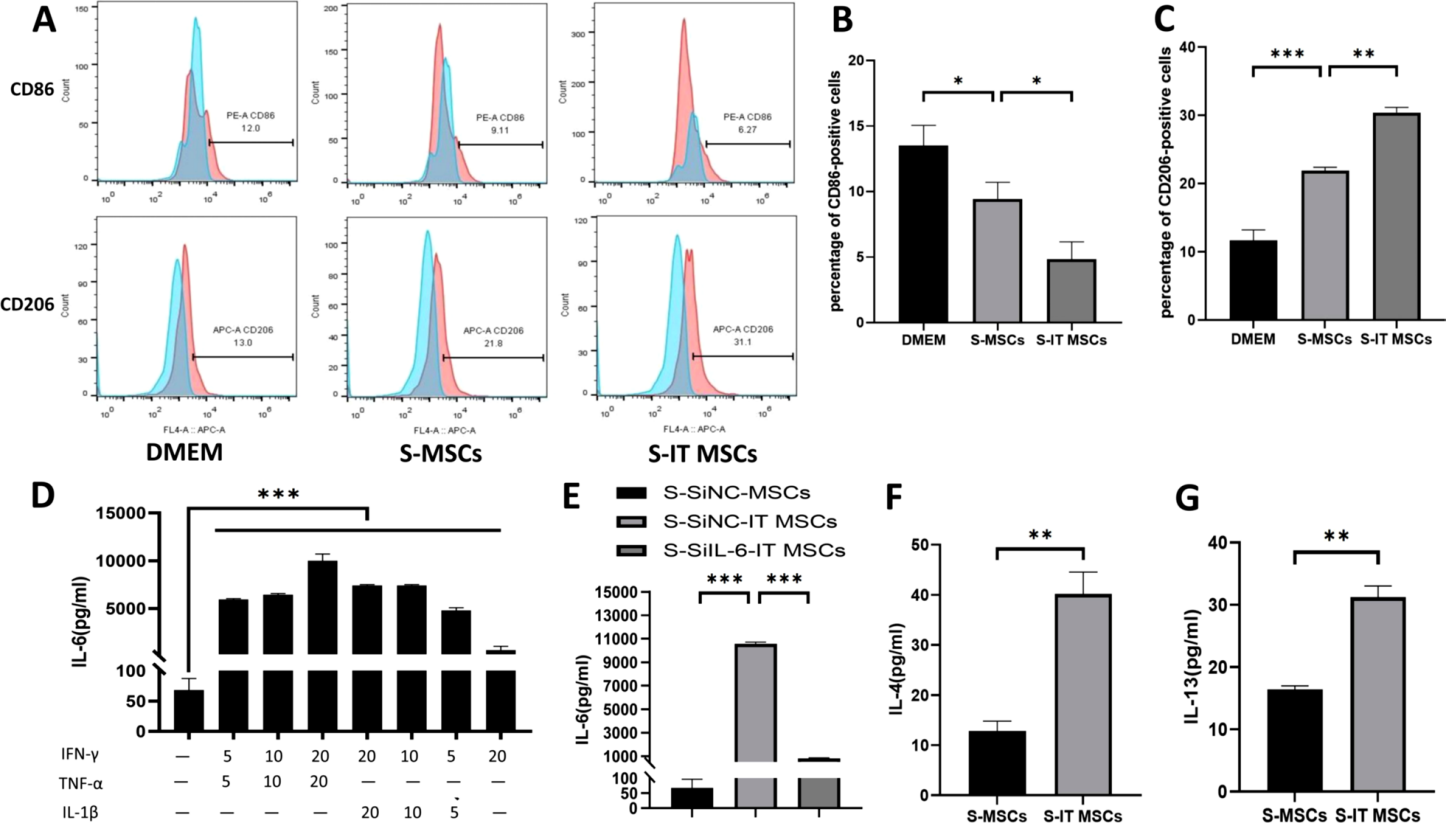
The S-IT MSCs promotes more efficient polarization of macrophages toward the M2 phenotype than the S-MSCs. Bone marrow-derived macrophages were cultured in M-CSF for 7 days followed by culture with DMEM, the S-MSCs and the S-IT MSCs for 24 h. Cultured cells were collected and immunostained with CD206-APC and CD86-PE followed by flow cytometry analysis. The concentrations of several cytokines in the S-MSCs and S-IT MSCs were determined by ELISA. **A**–**C** Percentage of CD206-positive and CD86-positive cells in macrophages after treatment with DMEM, S-MSCs, and S-IT MSCs by flow cytometry. **D** IL-6 secretion by MSCs after stimulation by various inflammatory factors. **E** IL-6 expression after IL-6 was silenced in IT MSCs using siRNA. **F**, **G** Expression of IL-4 and IL-13 in the S-IT MSCs. The results represent three independent experiments. *P < 0.05; **P < 0.001, and ***P < 0.0001

Our results showed that the number of CD206-positive macrophages was significantly higher in the S-IT MSCs-treated group than in the S-MSCs- or DMEM-treated group, whereas the trend was reversed for the number of CD86-positive macrophages (Fig. 1A–C). This indicated that S-IT MSCs are more effective than S-MSCs in promoting the polarization of macrophages toward M2.

It has been found that IL-6 is upregulated in MSCs stimulated with proinflammatory cytokines [29]. IL-6 is one of the most important factors in immunomodulation because it promotes polarization toward the M2 phenotype [20, 30]. In this study, we used ELISA to detect different types and levels of acute inflammatory factors to pretreat MSCs and found that the increment of IL-6 secreted by MSCs stimulated with 20 ng/mL each of IFN-γ and TNF-α was the most significantly (Fig. 1D).

Moreover, studies have shown that IL-6 exerts immunomodulatory effects by upregulating IL-4Rα receptor expression on the surface of macrophages, promoting IL-4 and IL-13 binding to this receptor, activating the downstream STAT6/PPARγ signaling pathway, promoting macrophage polarization toward the M2 phenotype ultimately [30, 31]. Next, we used ELISA to detect the expression of IL-4 and IL-13 by IT MSCs. Our result showed that the ability of MSCs to express IL-4 and IL-13 was also enhanced by IT stimulation (Fig. 1F, G).

Based on these above results, we propose the hypothesis that IT MSCs can promote rapid and high-quality wound healing by promoting macrophage polarization toward M2 through high expression of IL-6. To verify this hypothesis, we used small interfering RNA to silence the expression of IL-6 in IT MSCs, and we used ELISA to detect the silencing efficiency before proceeding with further exploration (Fig. 1E).

### IT MSC-secreted IL-6 plays a major role in macrophage phenotype remodeling

**Fig. 2:**
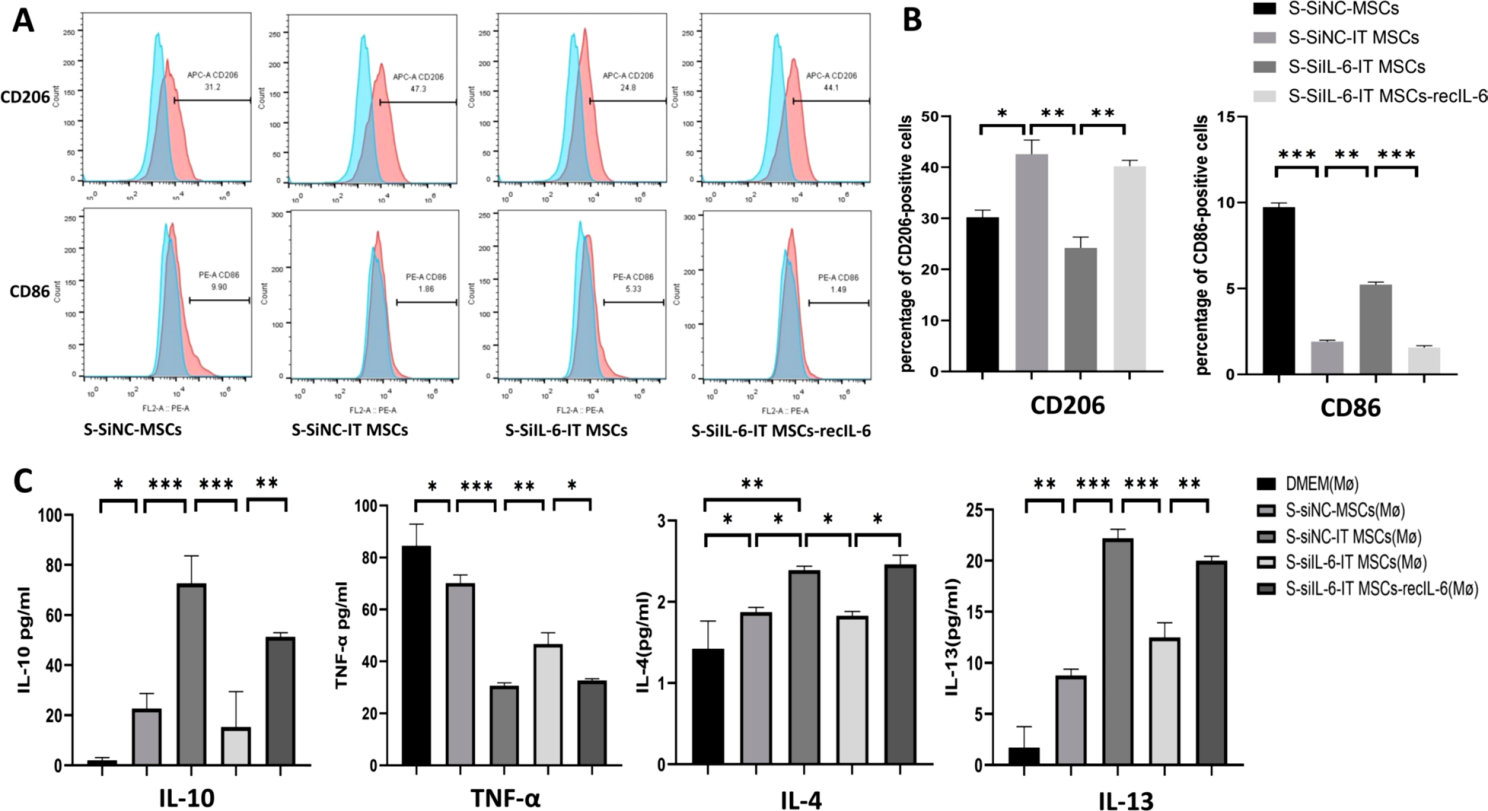
Silencing IL-6 inhibits IT MSCs-mediated induction of M2 macrophages. Macrophages were cultured with the S-siNC-MSCs, S-siNC-IT MSCs, S-siIL-6-IT MSCs (the supernatant of IT MSCs after IL-6 knockdown), and S-siIL-6-IT MSCs-recIL-6 (the supernatant derived from siIL-6-IT MSCs after adding 100 ng/mL recombinant IL-6). **A**, **B** The percentage of CD206-positive and CD86-positive cells in macrophages after treatment with the above supernatant groups according to flow cytometry. **C** The secretion of IL-10, IL-4, IL-13, and TNF-α in the supernatants of each group of MSCs-treated macrophages was determined using ELISA. Data are representative of three independent experiments. *P < 0.05; **P < 0.001, and ***P < 0.0001

To determine whether IL-6 is involved in IT MSCs-mediated polarization of M2 macrophages, we silenced the expression of IL-6 in IT MSCs with siRNA. The FCM and ELISA results showed that supernatant derived from IT MSCs that knockout IL-6 (S-SiIL-6-IT MSCs) no longer possessed an effect on promoting polarization toward the M2 phenotype. However, supplementation with recombinant IL-6 (100 ng/mL) restored this effect of the S-SiIL-6-IT MSCs in vitro (Fig. 2A, B). Furthermore, the S-siNC-IT MSCs and S-SiIL-6-IT MSCs-recIL-6 increased the secretion of IL-10, IL-4, and IL-13 but decreased the secretion of TNF-α by macrophages (Fig. 2C). These results indicated that IT MSCs promote macrophage polarization to the M2 phenotype through high expression of IL-6.

**Fig. 3:**
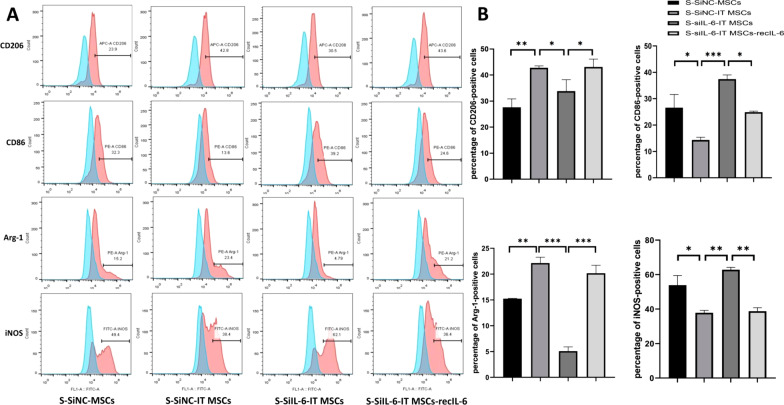
IL-6 secreted by IT MSCs is a major cytokine in remodeling the M1 phenotype to the M2 phenotype. Macrophages were prestimulated with 200 ng/ml LPS for 2 h to polarize them toward the M1 phenotype followed by the addition of supernatants from each group of stem cells and continued coincubation for 24 h. **A**, **B** The percentage of CD206- and Arg-1-positive as well as CD86- and iNOS-positive cells in M1 phenotype macrophages after treatment with the above supernatant groups according to flow cytometry. Data are representative of three independent experiments. *P < 0.05; **P < 0.001; ***P < 0.0001

To identify the potential ability of IT MSCs to remodel the M2 phenotype, we used 200 ng/ml LPS to prestimulate macrophages for 2 h to polarize them toward the M1 phenotype followed by coincubation with the supernatants from each group of stromal cells for an additional for 24 h. CD206, Arg-1, CD86, and iNOS were detected by FCM to investigate the M1 and M2 phenotypes. The FCM results revealed that the S-SiNC-IT MSCs and S-SiIL-6-IT MSCs-recIL-6 groups significantly increased the expression of CD206 and Arg-1 (the M2 phenotype marker proteins), but decreased the expression of CD86 and iNOS (the M1 phenotype marker proteins), compared to the S-SiNC-MSCs and S-SiIL-6-IT MSCs groups (Fig. 3A, B). Thus, these findings demonstrated that IT MSCs remodel the M1 phenotype to M2 via their high secretion of IL-6 and that this remodeling is superior to that of MSCs and DMEM.

### IL-6 enhances the phagocytic and angiogenic ability of IT MSCs by promoting polarization of M2 macrophages in vitro

Macrophage loss or depletion can lead to delayed healing or even nonhealing due to impaired clearance of cells debris from the wound [23]. M1 macrophages are present in large numbers during the early inflammatory response to trauma repair and produce large amounts of proinflammatory factors. In contrast, M2 macrophages predominate during the resolution phase, secreting mainly anti-inflammatory and growth factors, and they have high phagocytic activity, allowing them to remove necrotic and damaged cells from the trauma surface [5].

**Fig. 4:**
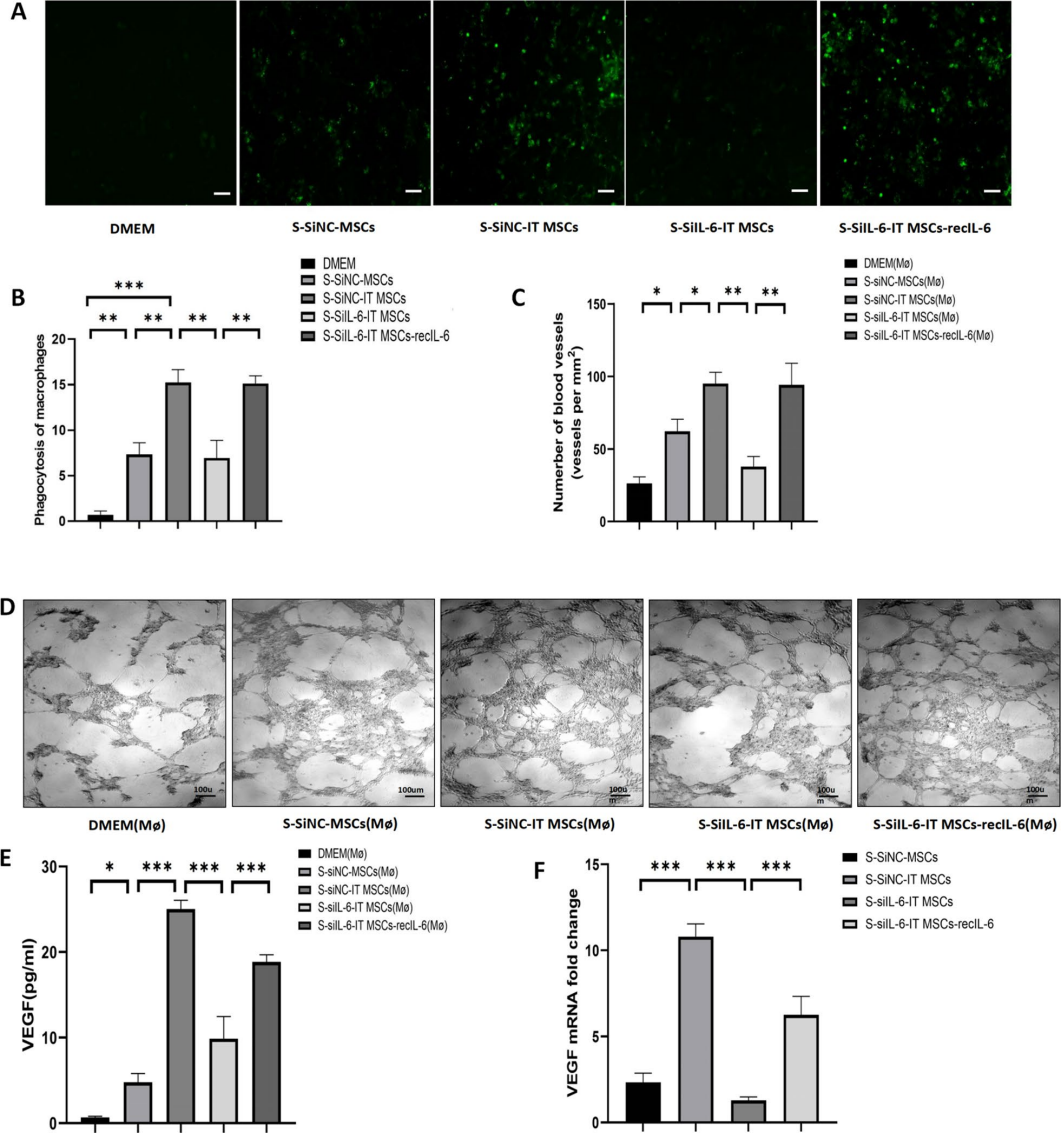
IL-6 enhances the phagocytic and angiogenic ability of M2 macrophages. **A**, **B** Immunofluorescence staining of M2 macrophages endocytosed with FITC-labeled dextran (green). **C** Quantitative analysis of the number of nodes using ImageJ software in the angiogenesis assay. **D** Representative images of the tube network in the angiogenesis assay. Images were acquired at 12 h. **E** The concentration of VEGF derived from macrophages pretreated with the supernatants of each group of stem cells was detected by ELISA. **F** VEGF gene expression of wound tissue on day 3 in vivo, n = 4 mice for each group. Data are representative of three independent experiments. *P < 0.05; **P < 0.001, and ***P < 0.0001. Scale bar 100 = μm

First, we detected whether the supernatant from IT MSCs has a greater effect in improving the phagocytic activity of M2 macrophages via high levels of IL-6. Our results showed that the S-SiNC-IT MSCs and S-SiIL-6-IT MSCs-recIL-6 treatments significantly enhanced the phagocytosis of FITC-labeled dextran by macrophages compared to the S-SiIL-6-IT MSCs, S-SiNC-MSCs, and DMEM treatments (Fig. 4A, B). Thus, these findings indicated that the supernatant from IT MSCs promotes phagocytosis of M2 macrophages in an IL-6-dependent manner and that this effect was superior to that of the S-MSCs and DMEM.

Next, we explored whether each group of stromal cell supernatants can play a role in improving endothelial cell function by promoting macrophage converting to the M2 phenotype in vitro. We cultured 3 × 10^4^ HUVECs on Matrigel with supernatant from macrophages after treatment with the supernatant of stromal cells in each group. The number of blood vessels predicted that endothelial cells capacity in formation of capillary-like structures was greatly enhanced by the S-SiNC-IT MSCs (Mø) compared to the DMEM (Mø) and S-SiNC-MSCs (Mø) in vitro. Moreover, the effect of the S-SiNC-IT MSCs (Mø) on HUVECs was largely abrogated by siRNA knockdown of IL-6, while the effect was rescued after treatment with the S-SiIL-6-IT MSCs-recIL-6 (Mø) in vitro (Fig. 4C, D).

Finally, to identify the potential mechanism underlying the above function in vitro, we collected supernatant from macrophages after prestimulation with the supernatant of stromal cells in each group and measured the concentration of VEGF in macrophage supernatants by ELISA. The ELISA results showed that the VEGF concentration in the S-SiNC-IT MSCs (Mø) was significantly higher than that in DMEM (Mø) and the S-SiNC-MSCs (Mø). Furthermore, the VEGF concentration in the S-SiIL-6-IT MSCs (Mø) was significantly reduced, while the VEGF concentration in the S-SiIL-6-IT MSCs-recIL-6 (Mø) was significantly restored in vitro (Fig. 4E). Moreover, the qPCR results of the in vivo trauma tissue confirmed the same trend in the expression of VEGF (Fig. 4F).

The above results were consistent with the trend of promoting macrophage polarization to the M2 phenotype by the stromal cell supernatant from each group, implying that the IT MSCs supernatant improves endothelial cell tube-forming function by promoting the polarization of macrophages to the M2 phenotype with high VEGF expression.

### IT MSC-secreted IL-6 promotes polarization of M2 macrophages via an IL-6-dependent signaling pathway.

STAT3 and STAT6 are key transcription factors that induce polarization of macrophages toward the M2 phenotype. Moreover, IL-6 has been reported to promote IL-4 and IL-13 binding to IL-4Ra receptors through upregulation of IL-4Ra expression on the surface of macrophages, which in turn activates the STAT6/PPARγ signaling pathway and ultimately promotes M2 polarization [30]. In addition, studies have found that IL-6 promotes macrophage polarization to the M2 phenotype via activates IL-6/STAT3 signaling pathway, which is inhibited in M1 macrophages but activated in M2 macrophages [31, 32].

**Fig. 5:**
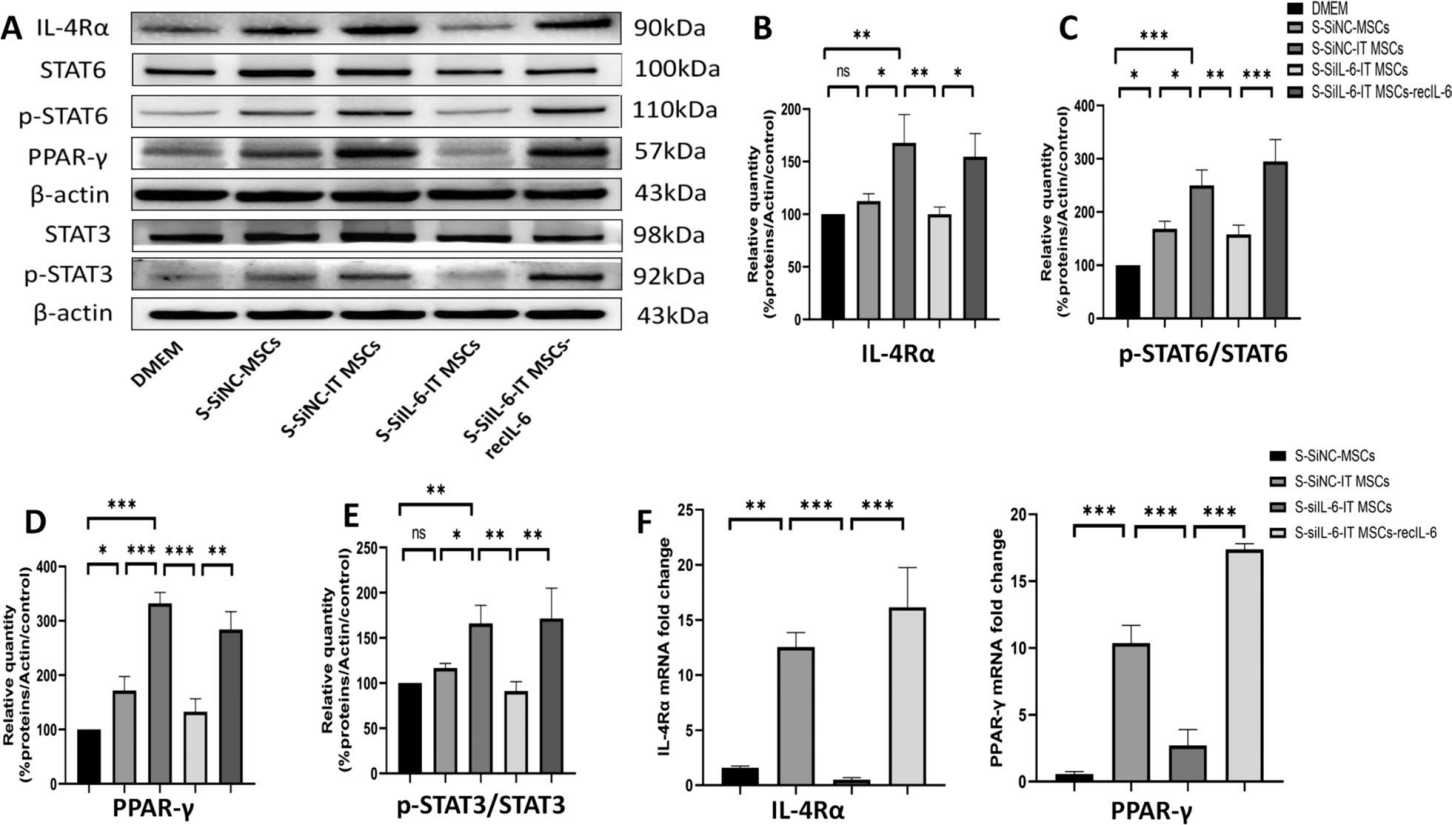
IL-6 secreted by IT MSCs promotes polarization of M2 macrophages via an IL-6-dependent signaling pathway. **A** Western blot analysis of IL-4Rα, STAT6, p-STAT6, PPAR-γ, STAT3, and p-STAT3 expression. **B**–**E** Corresponding histogram protein levels or ratios of IL-4Rα, p-STAT6/STAT6, PPAR-γ, and p-STAT3/STAT3. **F** IL-4Rα and PPAR-γ gene expression of wound tissue on day 3 in vivo, n = 4 mice for each group. Data are representative of four independent experiments. *P < 0.05; **P < 0.001, and ***P < 0.0001

Thus, we next explored whether IT MSCs supernatants promote macrophage converting to the M2 phenotype through the IL-6/IL-4Rα/STAT6/PPAR-γ and IL-6/STAT3 signaling pathways by Western blot analysis in vitro (Fig. 5). In comparison with the S-SiIL-6-IT MSCs, we observed higher levels of IL-4Rα and PPAR-γ expressed by macrophages treated with the S-SiNC-IT MSCs. Moreover, exogenous IL-6 protein rescued this trend. The STAT3 and STAT6 were phosphorylated after treated with the S-SiNC-IT MSCs. However, silencing of IL-6 using siRNA in IT MSCs decreased the phosphorylation of STAT3 and STAT6, but this effect was restored after the exogenous addition of 100 ng/mL IL-6 (Fig. 5A).

The ratios of p-STAT3/STAT3 and p-STAT6/STAT6 as well as the levels of IL-4Rα and PPARγ were more pronounced in macrophages in the S-SiNC-IT MSCs and S-SiIL-6-IT MSCs-recIL-6 groups but reduced in the S-siIL-6-IT MSCs group. Our results indicated that more IL-6 was produced by MSCs after stimulated with IT, which promotes macrophage remodeling to the M2 phenotype via the IL-6/IL-4Rα/STAT6/PPAR-γ and IL-6/STAT3 signaling pathways (Fig. 5B–E). The qPCR results for the in vivo trauma tissue reconfirmed the same trend in the expression of IL-4Rα and PPAR-γ as well as the key genes in the IL-6-dependent signaling pathway (Fig. 5F).

The above results suggested that the S-IT MSC-mediated macrophage polarization toward the M2 phenotype depends on IL-6, mainly through the IL-6/IL-4Rα/STAT6/PPARγ and IL-6/STAT3 signaling pathways.

### IL-6 enhances cutaneous wound healing in mice

**Fig. 6:**
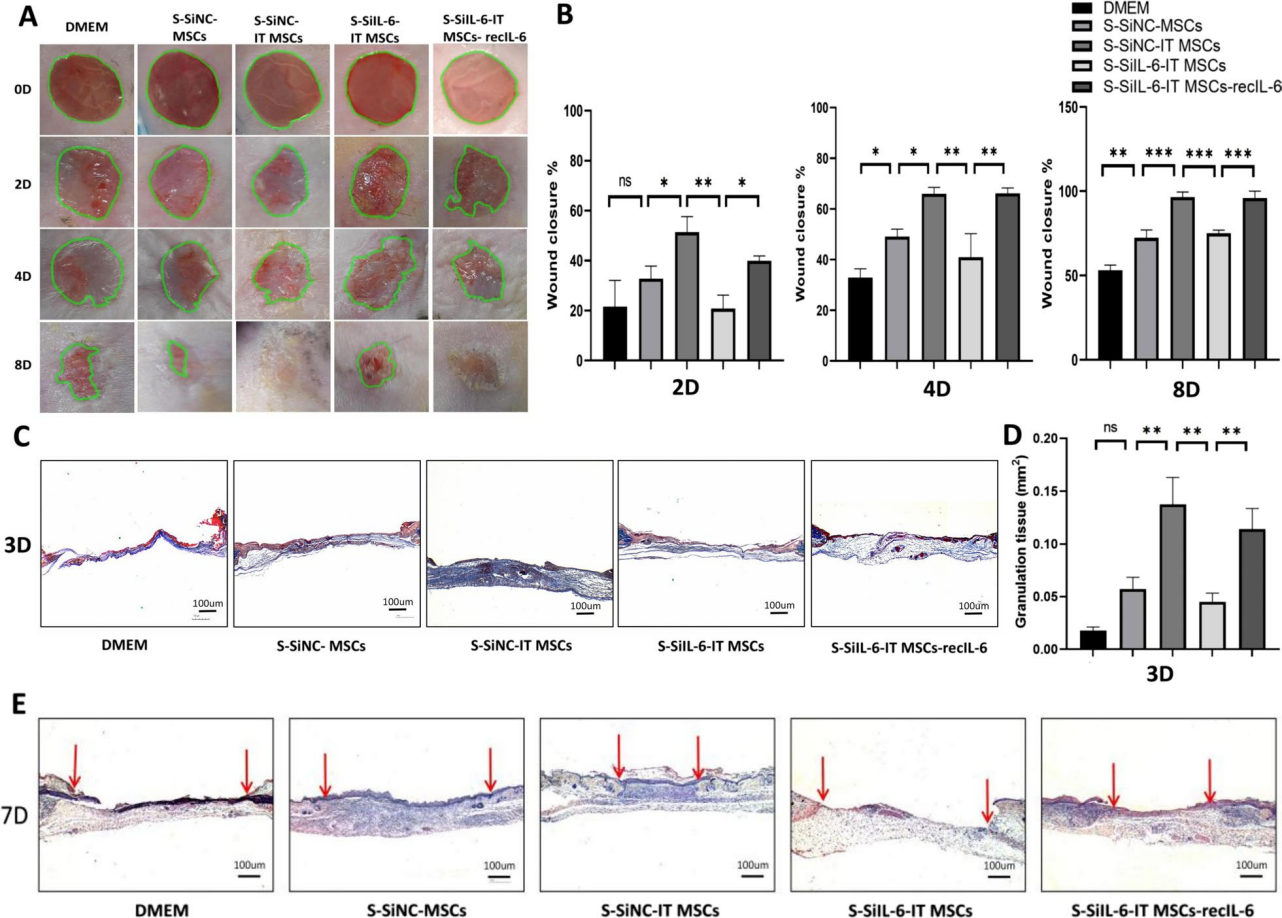
IL-6 enhances cutaneous wound healing in mice. **A** Gross view of wounds in the DMEM, S-siNC-MSCs, S-siNC-IT MSCs, S-siIL-6-IT MSCs, S-siIL-6-IT MSCs-recIL-6 treatment groups at days 0, 2, 4, and 8 post administration. **B** The rate of wound closure in wounds receiving different treatments at the indicated times. **C** Representative images of Masson’s trichrome staining after wound treatment at day 3. The granulation tissue (blue) is characterized by a large amount of collagen deposition. Muscle under the wounds is stained red. Scale bar = 100 µm. **D** Quantification of granulation tissue thickness. **E** H&E staining of wound sections at day 7 post administration. Red arrows indicate tips of the epithelium tongue. Data are representative of four independent experiments. Scale bar = 100 μm. n = 4 mice for each group, *P < 0.05; **P < 0.001; and ***P < 0.0001

To explore the role of IL-6 in S-IT MSCs in promoting wound closure, we used small interfering RNA to silence IL-6 expression in MSCs, and the supernatant was collected after IT stimulation and condensed the supernatant to a tenth of its original volume by a 3-kDa ultrafiltration membrane. The excisional cutaneous wounds were then treated with the condensed supernatant. The results showed that the rate of wound closure was significantly lower in the S-SiIL-6-IT MSCs-treated group on day 2, day 4 and day 8 compared to the S-SiNC-MSCs-treated group. However, when recombinant IL-6 was added exogenously, the wound healing rate in the S-SiIL-6-IT MSCs-recIL-6 was comparable to that in the S-SiNC-IT MSCs group. In addition, compared to the DMEM group, the wound-healing rate was accelerated in all groups of stromal cell supernatant-treated wounds at days 2, 4, and 8 (Fig. 6A, B).

Timely coverage of the wound by granulation tissue is more conducive to the migration of surrounding epithelial cells to the wound bed, which is the key to accelerated wound repair. The Masson’s trichrome staining results showed that the blue collagen fraction in the S-SiNC-IT MSCs group was thicker than that in the S-SiNC-MSCs and DMEM groups on day 3, while this effect was attenuated when IL-6 was knocked down and restored with exogenous addition of recombinant IL-6 (Fig. 6C, D). These results indicated that the supernatant of IT MSCs promotes collagen deposition and earlier granulation tissue formation by modulating the polarization of the M2 phenotype in the early stage of wound healing, which aids the progression of re-epithelialization, thereby accelerating wound healing.

H&E staining also showed that the wound closure rate was reduced in the S-SiIL-6-IT MSCs treatment group compared with S-SiIL-6-IT MSCs, but the wound closure rate was significantly improved in the S-SiIL-6-IT MSCs-recIL-6 treatment group on day 7 as indicated by the red arrows (Fig. 6E). Therefore, IL-6 secreted by IT MSCs is a key factor in promoting a rapid wound healing.

### IL-6 increases the proportion of M2 phenotype in the wound tissue of mice

**Fig. 7:**
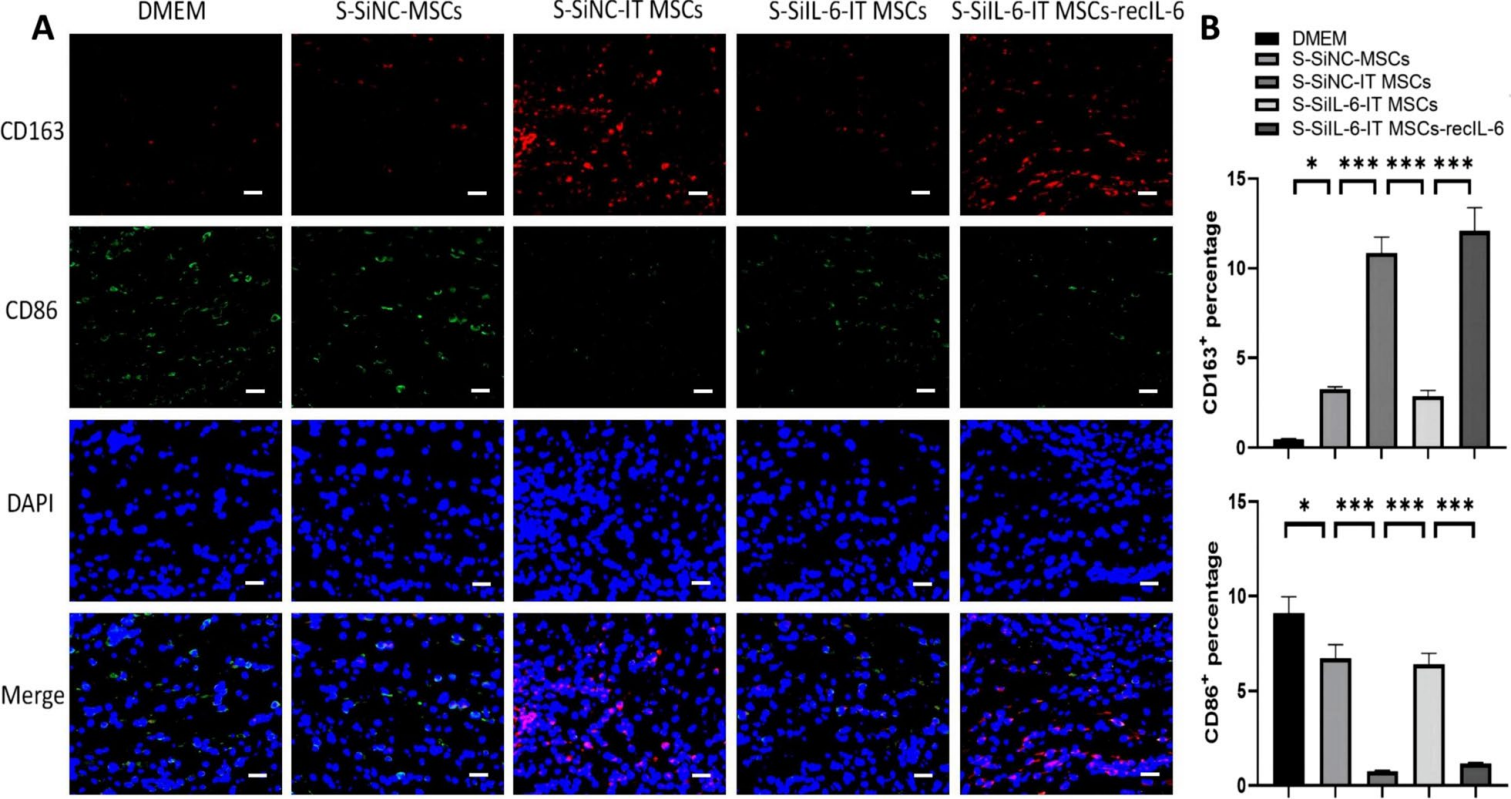
IL-6 secreted by IT MSCs increases the percentage of M2 macrophages via an IL-6-dependent signaling pathway in the local wounds of mice. **A** Immunofluorescence staining of CD86 (green), CD163 (red), and DAPI (blue) at the wound site after systemic injection of the DMEM, S-siNC-MSCs, S-siNC-IT MSCs, S-siIL-6-IT MSCs, S-siIL-6-IT MSCs-recIL-6 at day 3. **B** Corresponding histogram percentage of M1 and M2 in the local wounds. The numbers of stained nuclei were counted in three high-power fields per well under fluorescence microscopy. Data are representative of four independent experiments. n = 4 mice for each group, Scale bar = 20 μm, *P < 0.05; **P < 0.001, and ***P < 0.0001

We next investigated the in vivo effects of IT MSCs-derived-IL-6 on M2 polarization in wound bed tissue. In the present study, the CD163- and CD86-positive cells were considered as M2 and M1 phenotypes, respectively. The immunofluorescence results showed CD163-positive cells (red) in skin wounds were increased on day 3 after S-SiNC-IT MSCs treatment compared to those cultured with DMEM and S-SiNC-MSCs in addition to fewer CD163-positive cells in the IL-6 inhibitor group. More importantly, this effect was restored by exogenous addition of IL-6. The expression of CD86 (green) in skin wounds tended to be the opposite (Fig. 7A, B). These findings suggested that IT-MSCs are capable of promoting macrophages infiltrated at the wound sites convert to M2 phenotype, potentially contributing to modulating the inflammatory response and promoting wound repair.

### IL-6 mediates the proangiogenic ability of the IT MSC supernatant by promoting M2 polarization in macrophages in vivo

**Fig. 8:**
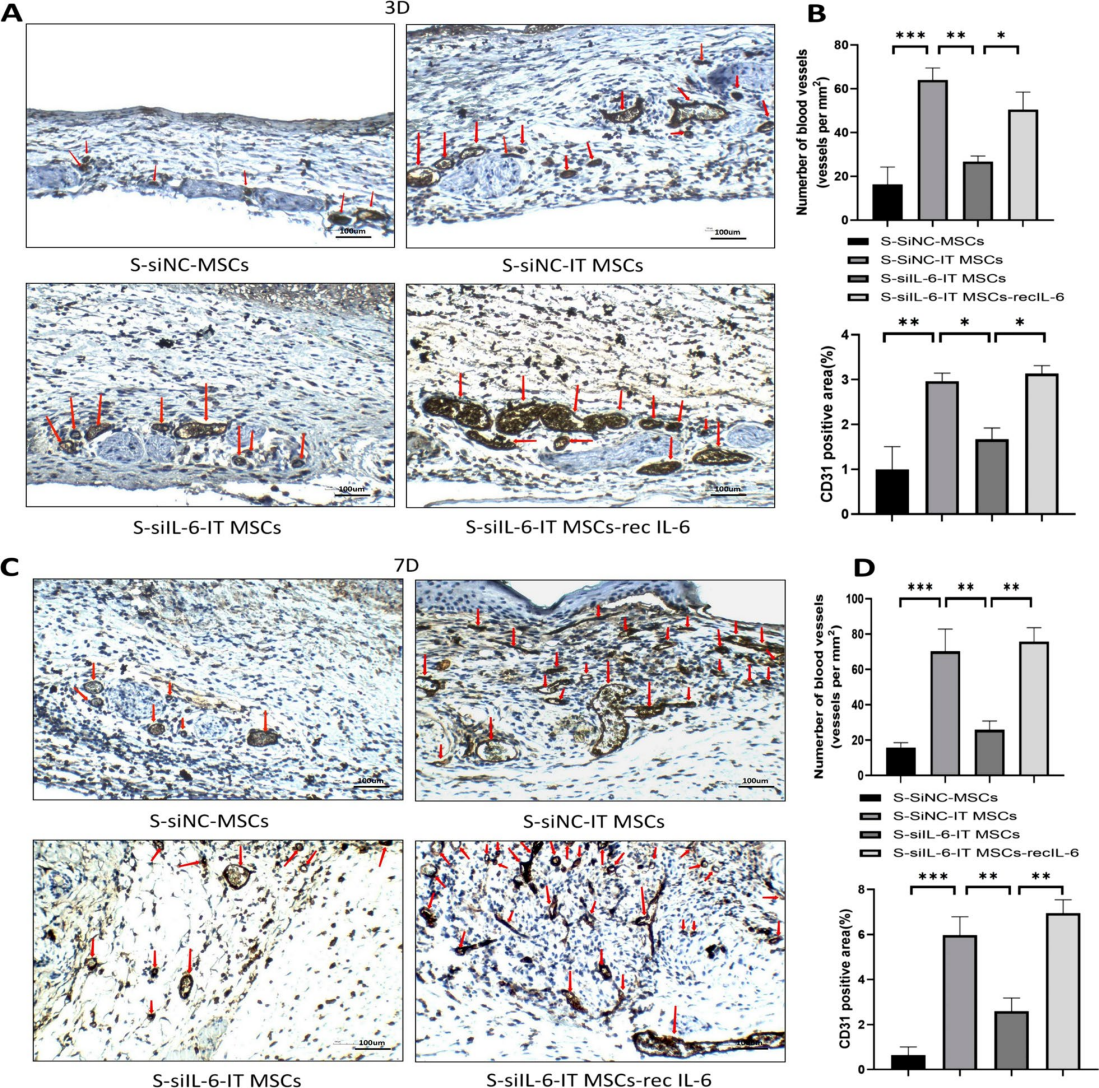
IT MSCs-derived IL-6 promotes angiogenesis at local wounds. **A**, **C** Cutaneous wounds on days 3 and 7 were stained with CD31 immunohistochemistry, and micrographs were acquired. Red arrows indicate blood vessels. **B**, **D** The number of blood vessels in each section was counted at the indicated time points, and the results are presented as the number of blood vessels per mm.^2^. CD31 positive area (%) was also detected by ImageJ. The numbers of stained CD31 were counted in four high-power fields per well under microscopy. Data are representative of four independent experiments. n = 4 mice for each group, Scale bar = 100 μm. *P < 0.05; **P < 0.001, and ***P < 0.0001

To confirm the effects of IT MSCs supernatant-treated macrophages on the function of vascularization in skin wounds, we used immunohistochemical methods to detect vascularization in the trauma by labeling neovascularization with CD31 on days 3 and 7. Wounds treated with S-SiNC-IT MSCs had significantly more tubules and CD31-positive areas in each field of view compared to wounds treated with S-SiNC-MSCs or DMEM. We found that the pro-angiogenic ability of S-SiNC-IT MSCs was lost in the absence of IL-6, but this effect could be largely restored when exogenous recombinant IL-6 was added (Fig. 8A, B). Furthermore, the S-SiNC-IT MSCs treatment resulted in highly increased vascularization of trauma tissue on day 7 compared to treatment with the S-SiIL-6-IT MSCs, and a similar increase was found in the S-SiIL-6-IT MSCs-recIL-6 treated group (Fig. 8C, D). Collectively, these results demonstrated that S-IT MSC-activated macrophages further enhance the function of endothelial cells in local wound tissue to form vessels compared to S-MSC-activated macrophages and that this effect is achieved via high expression of IL-6 by IT MSCs.

### IT MSC-secreted IL-6 optimizes the quality of the regenerated skin by promoting macrophage polarization toward M2

**Fig. 9:**
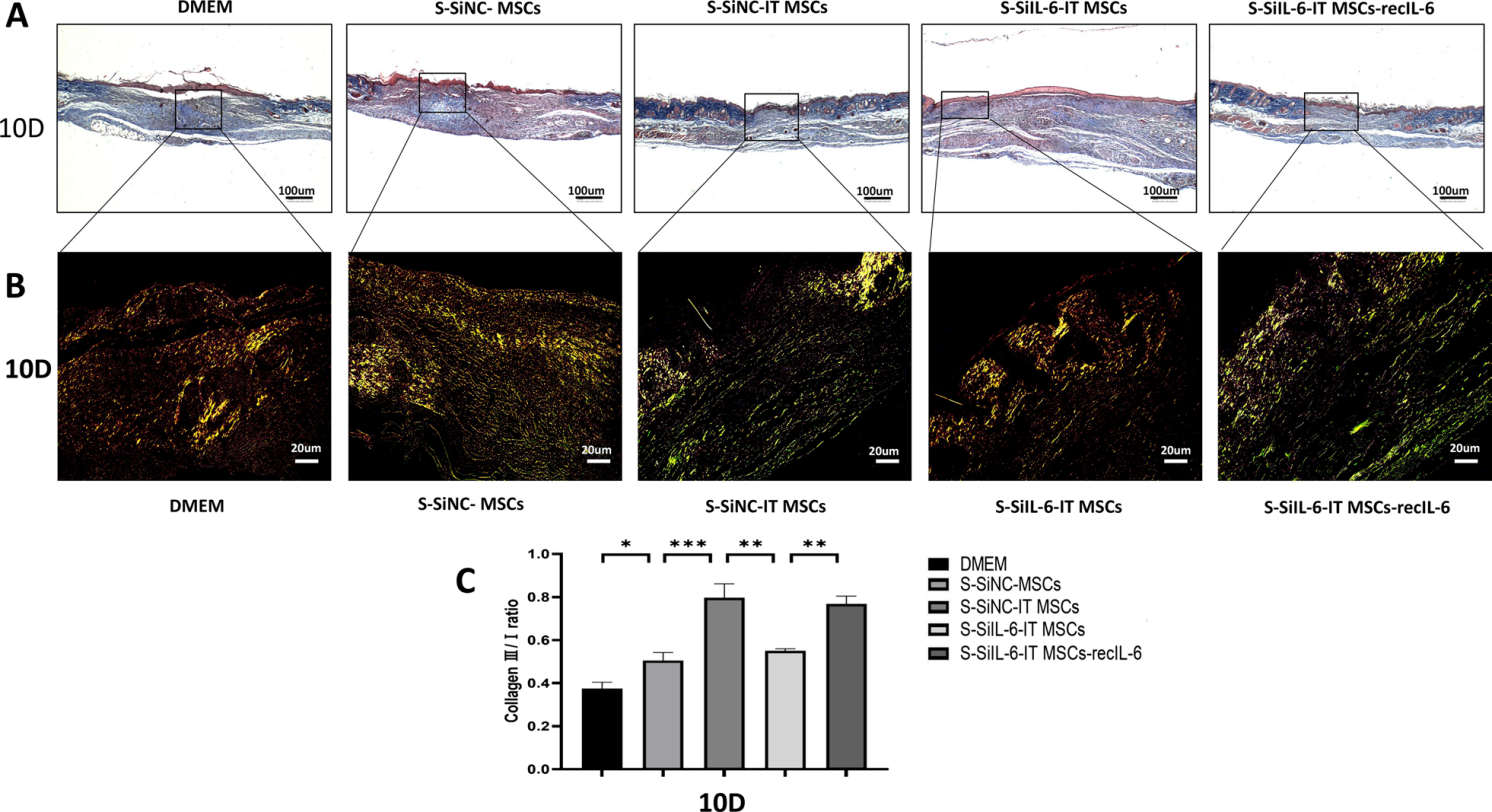
Posterior wound-healing assessment after 10 days. **A** Representative images of Masson’s trichrome staining. The thickness of collagen deposits (blue) represents the thickness of scar tissue after wound healing. **B** Representative images of picrosirius red staining. Type I collagen in the image is shown as orange or yellow fibers, and type III collagen is shown as green fibers when imaged with polarized light. **C** Quantification of the collagen type III/I ratio. Data are representative of four independent experiments. n = 4 mice for each group, Scale bar = 20 μm. *P < 0.05; **P < 0.001, and ***P < 0.0001

To evaluate the quality of wound healing, the thickness of collagen deposition and the ratio of type III/I collagen were observed by Masson’s trichrome staining and Sirius red staining, after 10 days of treatment of stromal cell supernatant in each group, respectively. Compared to the S-SiNC-MSCs or DMEM groups on day 10, the result of Masson’s trichrome staining showed that the thickness of the blue collagen fraction of new skin in the S-SiNC-IT MSCs group was closer to the normal skin thickness, while this effect was attenuated when IL-6 was knocked down and restored with exogenous addition of recombinant IL-6. In addition, the newly formed skins were thicker in the DMEM, S-SiNC-MSCs groups (Fig. 9A).

The ratio of type III/I collagen is often measured to characterize the scarless tissue of regenerated wounds [33]. Studies have found that excessive deposition of thick type I collagen makes the tissue hard and inelastic, causing scar hyperplasia; additionally, during the healing process of scarless wounds, the type III collagen content is higher in fetuses than in adults [33, 34]. In this study, we observed that IT MSCs-derived-IL-6 highly increased the ratio of type III/I collagen by promoting macrophage polarization toward the M2 phenotype, which reduced scar formation. When IL-6 was knocked out, this increased effect on the ratio of type III/I collagen in IT MSCs was diminished, while exogenous IL-6 restored this effect (Fig. 9B, C). Therefore, IL-6, which is highly expressed by IT MSCs, regulates inflammatory reactions in wounds by promoting macrophages convert toward the M2 phenotype, resulting in faster scarless skin regeneration.

## Discussion

The skin is the body's first line of defense against environmental exposure. Therefore, skin tissue integrity must be restored quickly to prevent infection and reduce fluid loss after injury [35, 36]. During the inflammatory phase of wound repair, macrophages play an important role in the onset and resolution of inflammation by reprogramming to adapt to changes in the microenvironment. In addition, during the proliferative phases, they also promote the reconstitution of vascular networks, facilitate collagen synthesis in the wound bed, and eventually form granulation tissue, providing a temporary matrix for cells to support migration, communication, and proliferation [37, 38]. M1 and M2 macrophages exert a key role in the different stages of wound repair, respectively [39–41]. Classically activated M1-type macrophages play a major pro-inflammatory role in wound repair and are characterized by TNF-α and iNOS [37]. Anti-inflammatory M2 cells regulate the entire repair process and secrete growth factors (VEGF, PDGF, and TGF-β1) that stimulate proliferation, angiogenesis, and fibroblast differentiation to myofibroblasts in the wound, ultimately regulating the scar outcome [42].

The stromal cells used in this study are from human umbilical cord (UC-MSCs). In clinical resources, umbilical cords are medical waste and are easier to obtain by noninvasive methods, and the MSCs isolated from umbilical cord Wharton's jelly have the highest purity and have higher proliferation capacity in vitro compared with those from other adult tissue. Moreover, the immunogenicity of UC-MSCs is lower than those from other adult tissue, while the paracrine effect is more potent [43–46]. Studies have shown that the bioactive factors in the supernatant from allogeneic or xenogeneic sources of stromal cells exert an equal effect on the therapeutic effect of the disease with little or no immune rejection aspect [7].

Numerous studies have showed that MSCs from different sources have the ability to promote wound repair [5, 47, 48]. However, studies have also shown that less than 1% of MSCs survive beyond one week after transplantation [49, 50]. Some researchers have demonstrated that paracrine of MSCs plays a key immunosuppressive role in wound healing; especially, MSCs-derived prostaglandin E2 (PGE2), NO, IL-6, and miR-223 were found to accelerate wound healing by promoting macrophage polarization toward the M2 phenotype [5, 20–22, 51]. Interestingly, studies have shown that the immunosuppressive effect of soluble factors secreted by MSCs is significantly enhanced after stimulation by acute inflammatory factors [7, 13, 29]. It has been shown that the ability of MSCs to secrete IL-6 is significantly enhanced when cocultured with lymphocytes, and MSCs exert immunosuppressive effects by promoting M2 polarization and partially suppressing T cell activity through high expression of IL-6, and this study also found that PGE2 exerted immunosuppressive effects which were also closely related to IL-6 [52]. It is known that IFN-γ and TNF-α are the mainly acute inflammatory factors secreted by lymphocytes. Interestingly, Xu et al. found that IFN-γ-based combined with certain concentrations of TNF-α or IL-1β pre-stimulated MSCs in vitro could enhance their effects of immunosuppression and promotion of wound healing via high expression of IL-6 and found that IFN-γ combined with TNF-α (10 ng/mL) pre-stimulated MSCs had the most significant increase in promoting IL-6 secretion from MSCs [13, 29]. Their further studies found that IFN-γ and TNF-α stimulate MSCs to secrete large amounts of IL-6, mainly through upregulation of the key transcription factor C/EBPβ [29]. In addition, Philipp et al. found that MSCs secreted high levels of NO, IL-6, and PGE2 after preconditioned with IL-1ß and IFN-ɣ, and their further research demonstrated that MSC-mediated macrophage polarization strongly depends on IL-6, whereas a minor role for NO and PGE2 [20]. In this study, we found that IFN-γ and TNF-α (20 ng/mL) stimulate MSCs which can more largely amplify the IL-6 secretion (Fig. 1D). Therefore, in this study, primary mesenchymal stromal cells were extracted from umbilical cord Wharton's jelly, and then their supernatant was extracted after stimulation with IFN-γ and TNF-α and applied on a mice total skin excision to investigate the effectiveness and related mechanisms of S-IT MSCs in promoting wound repair.

Our research found that the effect of the IT MSCs supernatant on promoting M2 polarization to promote beneficial wound repair was more significant than that of the MSCs supernatant (Figs. 1 and 6). However, the underlying mechanisms remain unclear. This research focused on exploring the potential mechanism of wound healing mediated by IT MSC-derived IL-6 as an promoter for M2 phenotype. In the present study, we found that the supernatant from IT MSCs more significantly enhances wound healing via converting macrophages to M2-type, through which promoting endothelial cell tube formation, collagen deposition, and macrophage phagocytosis. Furthermore, our results indicated that the IT MSCs supernatant promotes the polarization of macrophages to the M2 phenotype via the IL-6-dependent signaling pathway, thereby enhancing rapid and scarless healing of cutaneous wounds.

Activation of M2 has been shown to be associated with multiple transcription factors, among which STAT3 and STAT6 are the key transcription factors for M2 activation [23, 24]. Some shown that the ability of macrophages to transition to the M2 phenotype has been blocked after knockdown of STAT3 and STAT6 in mice and humans [53–55]. It has been shown that IL-6 promotes macrophage polarization toward the M2 phenotype mainly by activating STAT3 or STAT6 in two ways, respectively. One way is that IL-6 activates STAT6 by upregulating IL-4Rα receptor expression on the surface of macrophages and promoting IL-4 and IL-13 binding to IL-4Rα receptors, and then the phosphorylated STAT6 binds directly to PPAR-γ and promotes M2 polarization by initiating gene transcription [30]. In addition, it was also shown that IL-4Rα-STAT6 and PI3K-mTORC2 activation upregulates another key transcription factor, interferon regulatory factor-4 (IRF-4), which directly promotes macrophage polarization toward M2-type, and this pathway plays an important role in the process of glucose metabolism [56]. The other way is that IL-6 activates STAT3 by forming a polymer with IL-6R and gp130; phosphorylated STAT3 directly enters the nucleus to induce macrophage polarization to the M2 phenotype, and the IL-6/STAT3 signaling pathway is inhibited in M1 macrophages but activated in M2 macrophages [31].

In this study, we found that the expression of IL-6 by MSCs is largely amplified in the presence of IFN-γ and TNF-α, and the in vitro and in vivo studies found that IT MSCs-derived IL-6 provides a strong program signal for macrophages remodeling. Furthermore, on the one hand, we found that IL-6 indeed upregulated IL-4Rα receptor expression on the surface of macrophages.

Subsequently, STAT6 is activated and PPARγ is upregulated indeed, which is consistent with the IL-6/STAT6/PPARγ signaling pathway. On the other hand, IT MSC-derived IL-6 activated STAT3, which is consistent with the IL-6/STAT3 signaling pathway. Therefore, we concluded that high levels of IL-6 derived from S-IT MSCs promote macrophages toward M2 phenotype via an IL-6-dependent signaling pathway.

Of course, in the early stages of traumatic inflammation, acute inflammatory factors IFN-γ and TNF-α are secreted by M1-type macrophages, which can also stimulate the auto stromal cells, and the paracrine effect of stimulated auto stem cells is likewise enhanced, promoting macrophages to M2 polarization and exerting a pro-healing effect. However, the number of resident stromal cells is limited, and this mechanism plays an important role only if there are enough MSCs recruited to the trauma surface. So this needs to be further explored.

## Conclusions

In conclusion, we demonstrated for the first time that high levels of IL-6 derived from S-IT MSCs remodel macrophages toward M2 phenotype via an IL-6-dependent signaling pathway, which contributes to the improvement of immune microenvironment, vascularization, macrophage phagocytosis, collagen deposition, and tissue remodeling of wound. These findings further supported that compared with S-MSCs, S-IT MSCs have more beneficial bioactive factors for wound repair and are a more promising stromal cell-based therapies for faster and higher-quality wound repair.

## Supplementary Information

**Additional file 1. Fig. S1**: Identification of UC-MSCs and macrophages. (A) Results of flow cytometry identification of UCMSC surface markers: CD105+, CD29+, CD90+ and CD73+ > 95.00%; CD31+, CD34+, CD45+ and HLA-DR+ < 2.00%. (B) Flow cytometry results of the macrophages-specific protein F4/80: F4/80-positive cells > 95.00%.

**Additional file 2.** Western blot original result of IL-4Rα.

**Additional file 3.** Western blot original result of PPAR-γ.

**Additional file 4.** Western blot original result of STAT3.

**Additional file 5.** Western blot original result of STAT6.

**Additional file 6.** Western blot original result of β-actin 1.

**Additional file 7.** Western blot original result of β-actin 2.

**Additional file 8.** Western blot original result of p-STAT3.

**Additional file 9.** Western blot original result of p-STAT6.

## Data Availability

The relevant data supporting the findings of this study are all included in this article and in the attachment files.

## Abbreviations

DMEM: Dulbecco’s modified Eagle’s medium
FBS: Fetal bovine serum
S-MSCs: Supernatant of mesenchymal stromal cells
IT: IFN-γ and TNF-α
S-IT MSCs: Supernatant of mesenchymal stem cells pretreated with IT
IL-6: Interleukin 6
LPS: Lipopolysaccharide
HUVECs: Human umbilical vein endothelial cells
S-SiNC-MSCs: Supernatant derived from MSCs that were transferred with nonsilencing siRNA
S-SiNC-IT MSCs: Supernatant derived from IT MSCs that were transferred with nonsilencing siRNA
S-SiIL-6-IT MSCs: Supernatant derived from IT MSCs that were transferred with IL-6 silencing siRNA
S-SiIL-6-IT MSCs-recIL-6: Supernatant derived from IT MSCs that knockout IL-6 and then supplemented with recombinant IL-6
Møs: Macrophages
ELISA: Enzyme-linked immunosorbent assay
H&E: Hematoxylin-eosin staining
IHC: Immunohistochemistry
IF: Immunofluorescence
FCM: Flow cytometry
