# Supplementary figures and images for: Mesenchymal stromal cells pretreated with proinflammatory cytokines enhance skin wound healing via IL-6-dependent M2 polarization
Source: Stem Cell Res Ther. 2022 Aug 13;13:414. doi: 10.1186/s13287-022-02934-9 (PMC9375394; doi:10.1186/s13287-022-02934-9)

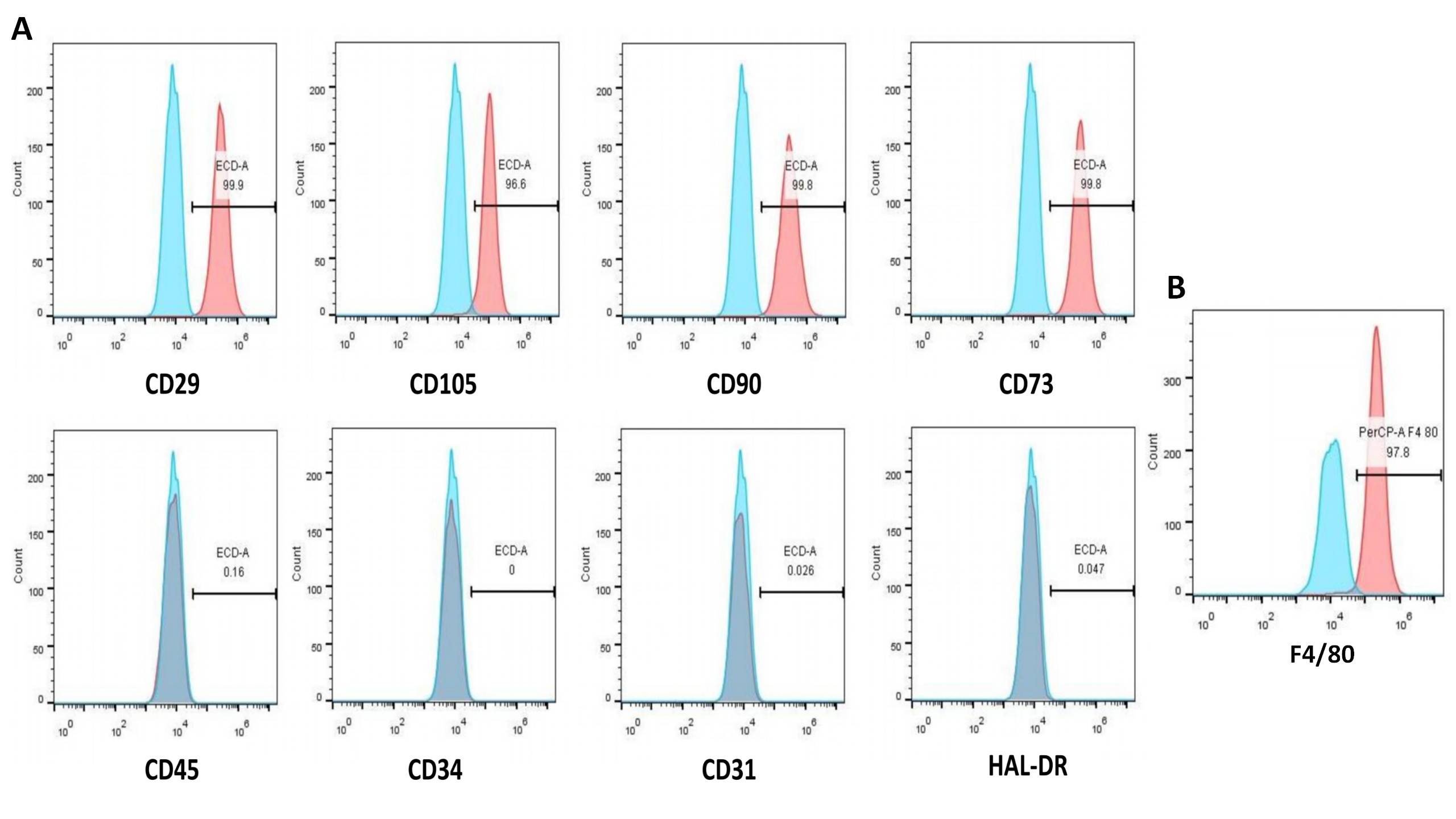

Supplement: Supplementary file 1 — Additional file 1. Fig. S1: Identification of UC-MSCs and macrophages. (A) Results of flow cytometry identification of UCMSC surface markers: CD105+, CD29+, CD90+ and CD73+ > 95.00%; CD31+, CD34+, CD45+ and HLA-DR+ < 2.00%. (B) Flow cytometry results of the macrophages-specific protein F4/80: F4/80-positive cells > 95.00%. [file 13287_2022_2934_MOESM1_ESM.tif]

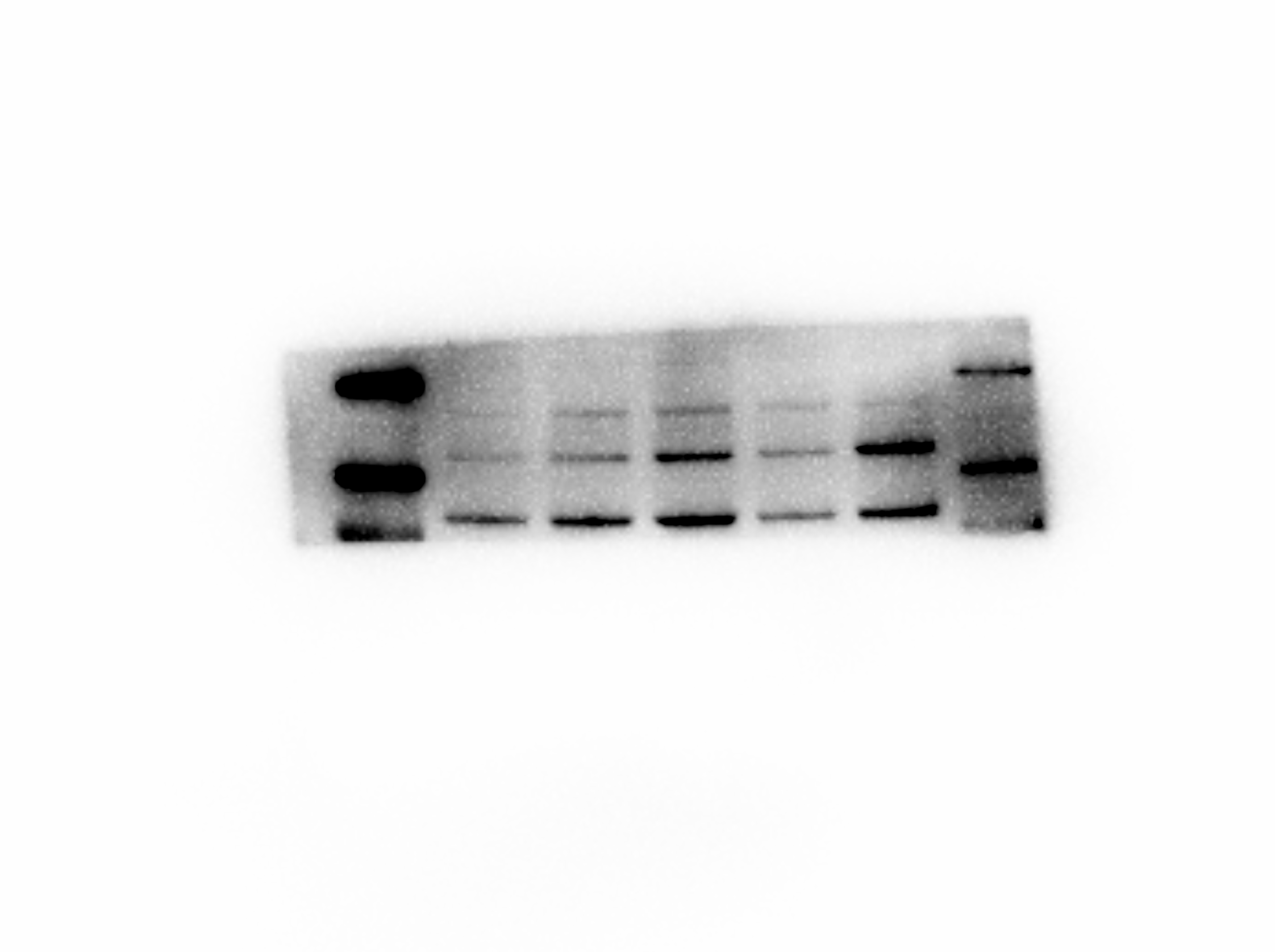

Supplement: Supplementary file 2 — Additional file 2. Western blot original result of IL-4Rα. [file 13287_2022_2934_MOESM2_ESM.tif]

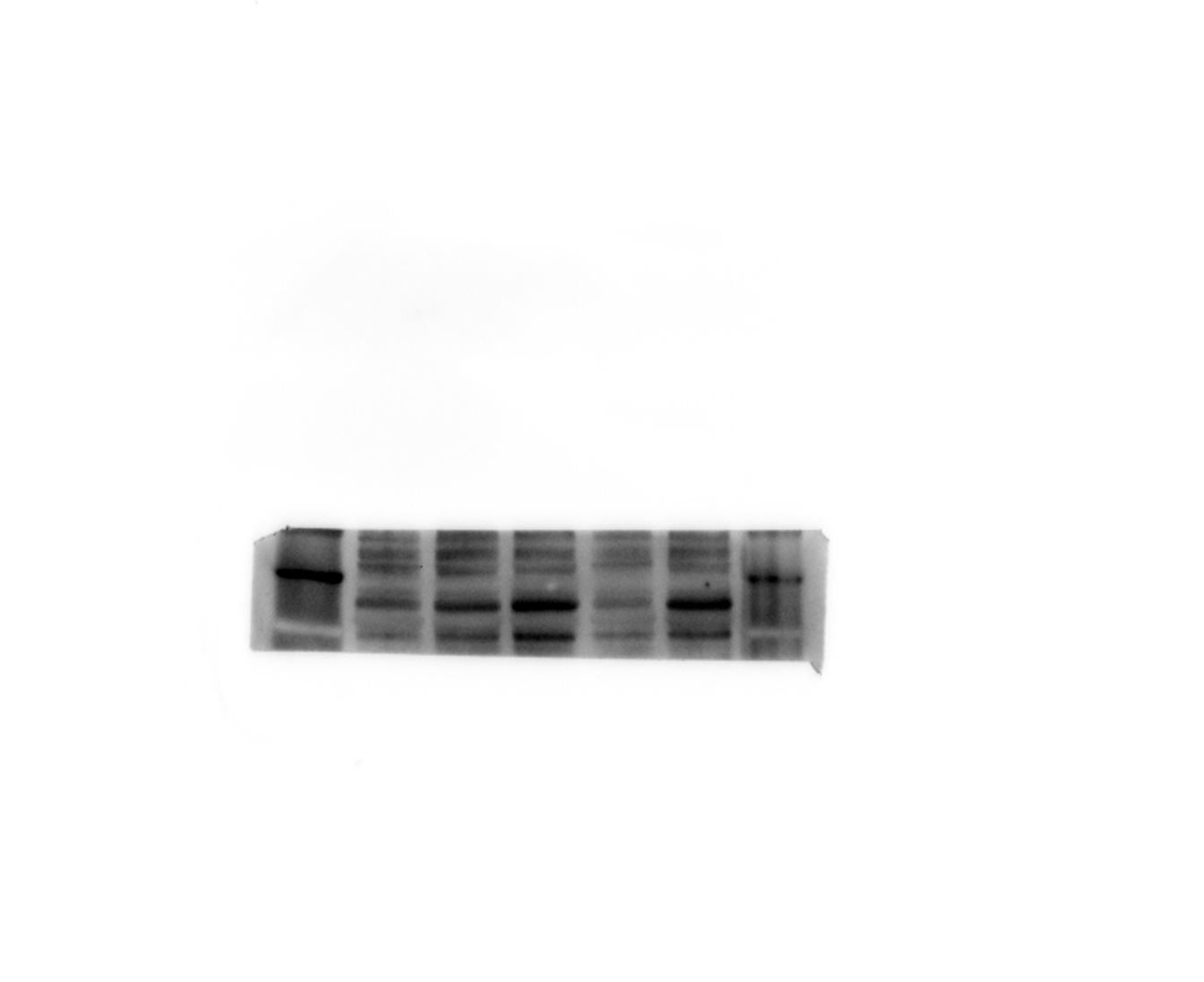

Supplement: Supplementary file 3 — Additional file 3. Western blot original result of PPAR-γ. [file 13287_2022_2934_MOESM3_ESM.tif]

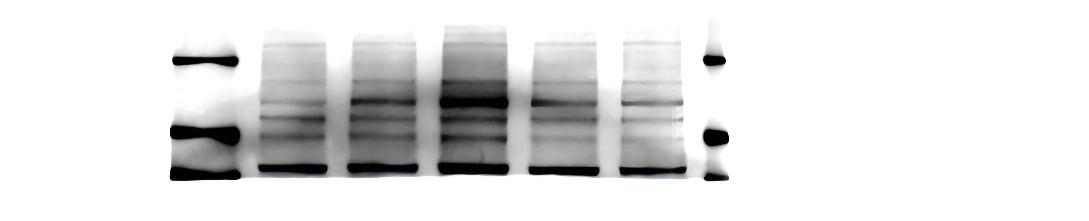

Supplement: Supplementary file 4 — Additional file 4. Western blot original result of STAT3. [file 13287_2022_2934_MOESM4_ESM.tif]

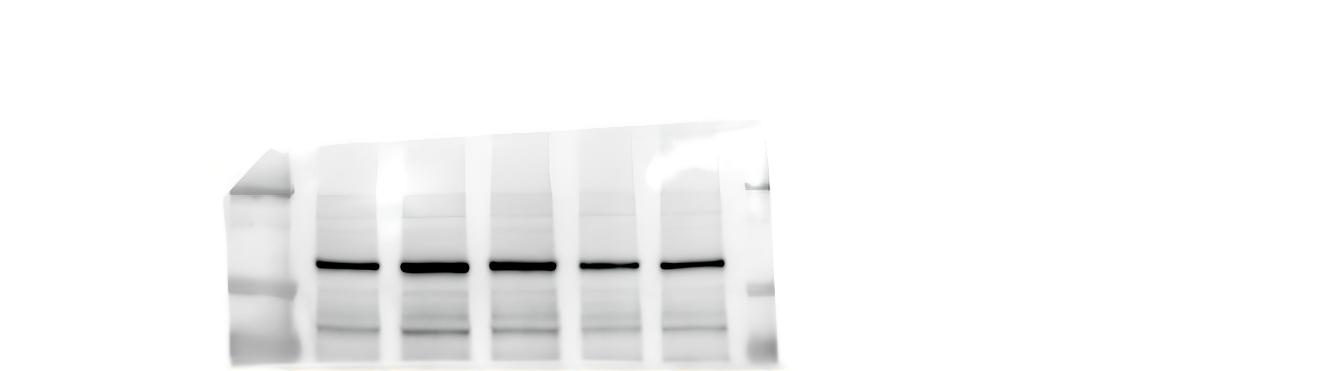

Supplement: Supplementary file 5 — Additional file 5. Western blot original result of STAT6. [file 13287_2022_2934_MOESM5_ESM.tif]

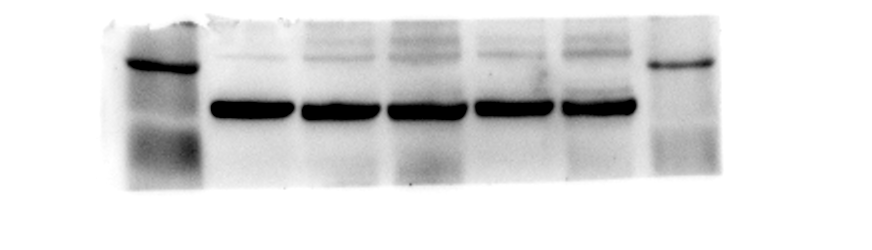

Supplement: Supplementary file 6 — Additional file 6. Western blot original result of β-actin 1. [file 13287_2022_2934_MOESM6_ESM.tif]

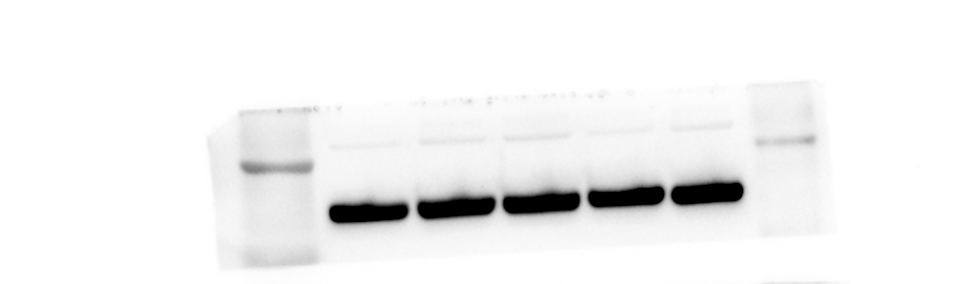

Supplement: Supplementary file 7 — Additional file 7. Western blot original result of β-actin 2. [file 13287_2022_2934_MOESM7_ESM.tif]

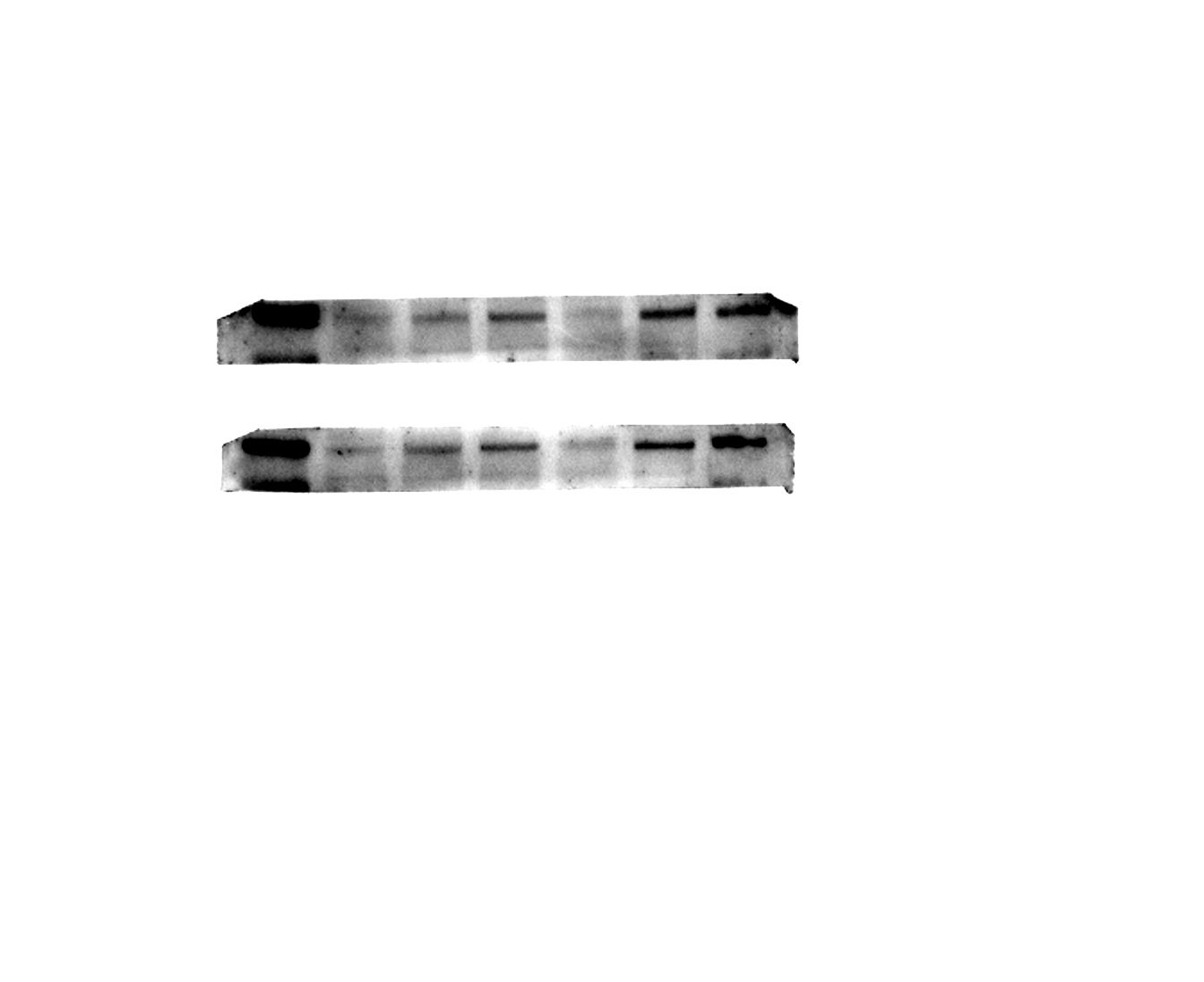

Supplement: Supplementary file 8 — Additional file 8. Western blot original result of p-STAT3. [file 13287_2022_2934_MOESM8_ESM.tif]

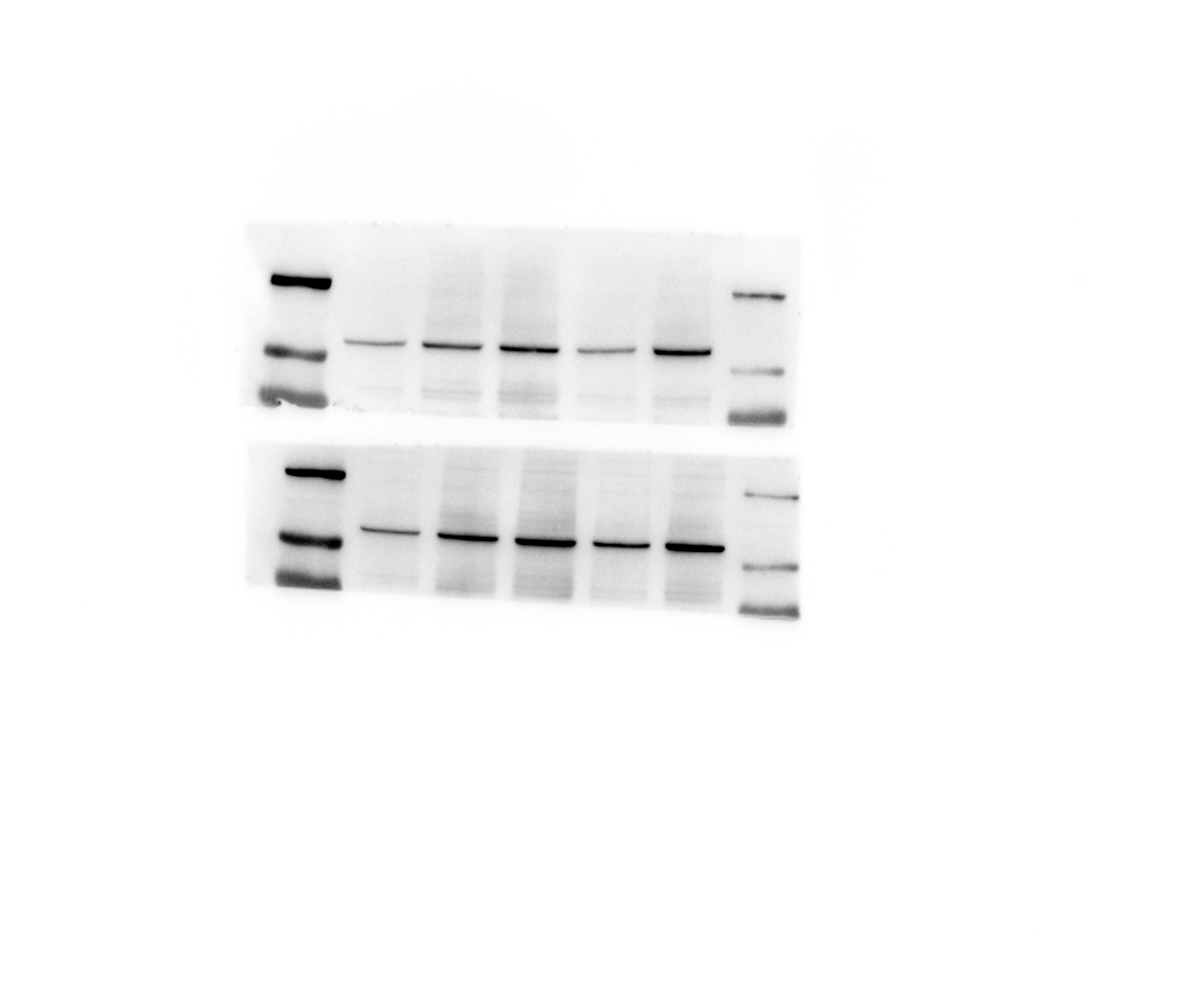

Supplement: Supplementary file 9 — Additional file 9. Western blot original result of p-STAT6. [file 13287_2022_2934_MOESM9_ESM.tif]
